# miR‐138‐5p inhibits proliferation and invasion in kidney renal clear cell carcinoma by targeting SINA3 and regulation of the Notch signaling pathway

**DOI:** 10.1002/jcla.23766

**Published:** 2021-09-29

**Authors:** Yang Liu, Hong‐chen Qu

**Affiliations:** ^1^ Department of Urological Surgery Liaoning Province Cancer Hospital & Institute (Cancer Hospital of China Medical University Shenyang China

**Keywords:** invasion, kidney renal clear cell carcinoma, miR‐138‐5p, Notch signaling pathway, proliferation, SIN3A

## Abstract

**Background:**

The function of miR‐138‐5p as an oncogenic factor has been reported in certain cancers. This study was performed to analyze the potential involvement of miR‐138‐5p in kidney renal clear cell carcinoma (KIRC).

**Methods:**

The Cancer Genome Atlas (TCGA) database was used to explain the expression of miR‐138‐5p in cancer and paired non‐cancer tissues of KIRC patients. Subsequently, miR‐138‐5p expression in KIRC tissues and cell lines, as well as that in normal tissues and normal renal tubular epithelial cell line, was detected. Artificial overexpressing of miR‐138‐5p was applied to observe its effect on the biological behaviors of KIRC cells. The target mRNA of miR‐138‐5p, SIN3A, was predicted and validated. Altered expression of miR‐138‐5p and SIN3A was introduced to confirm their functions in KIRC proliferation and invasion.

**Results:**

We showed that miR‐138‐5p was down‐regulated in tumor tissues of KIRC patients comparing to adjacent healthy tissues and linked to dismal prognosis in patients. miR‐138‐5p could hinder KIRC proliferation and invasion, while artificial overexpression of SIN3A led to reversed trends. SIN3A was a target mRNA of miR‐138‐5p. miR‐138‐5p and SIN3A together affect the activation of the Notch signaling pathway.

**Conclusion:**

This study evidenced that up‐regulated miR‐138‐5p inhibits proliferation and invasion of KIRC cells involving the transcription of SIN3A and the following regulation of the Notch signaling pathway.

## Graphical abstract

Bioinformatics analysis and molecular biology experiments were found and demonstrated that miR‐138‐5p is down‐regulated in kidney renal clear cell carcinoma (KIRC) and associated with proliferation and invasion in KIRC cells. miR‐138‐5p targeting SINA3 participates in the malignant phenotype of KIRC. This mechanism is implemented through the NOTCH pathway.

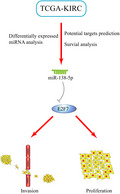

## 1 INTRODUCTION

According to the National Cancer Institute, there were about 73.8 thousand new kidney cancer cases in the USA, accounting for 4.2% of all new cancer cases and about 14.7 thousand deaths in 2019.^1^ The risk of kidney cancer is about 1 in 63. Renal cell carcinoma (RCC) occupies 85% proportion in kidney cancer. Especially, kidney renal clear cell carcinoma (KIRC) is the most common type of RCC.^2^ Surgery is the primary therapeutic regimen of KIRC. However, despite surgical treatment, at least 30% of patients with local tumors eventually relapse or metastasize.^3^ Hence, it is required to probe the underlying mechanisms and find a moving target of KIRC treatment.

miRNAs are endogenous noncoding small RNAs which are 19–25 nucleotides (nt) long and closely related to a variety of physiological processes.^4^, ^5^ Commonly, miRNAs could bind to the 3′‐untranslated regions (3′‐UTR) of mRNA, thereby inhibiting translation or activating transcription of target genes. Therefore, miRNAs play a crucial role in tumor progression: cell proliferation, migration, invasion, and apoptosis.^6^, ^7^, ^8^ Jia Lyu *et al*. introduced that the overexpressed miR‐222‐3p could inhibit KIRC metastasis by regulated TMP2 and ERK.^9^ miR‐206 inhibited cell proliferation of RCC by targeted ZEB2 and associated with the poor survival of RCC.^10^ miR‐122 acted as an onco‐miRNA to induce RCC proliferation and invasion.^11^

In the present research, we obtained the sequencing data and corresponding clinical data of KIRC from The Cancer Genome Atlas (TCGA) database to avoid bias. miR‐138‐5p was weak‐expressed in KIRC samples than in normal samples and associated with the poor survival of KIRC. Moreover, we found that SIN3 transcription regulator family member A (SIN3A) might be a target gene of miR‐138‐5p, and the expression of both was negatively correlated in clinical samples. Further data showed that SIN3A inhibited KIRC cell proliferation by Notch signaling pathway. In all, our data implied that miR‐138‐5p could be a delight target for KIRC therapy.

## 2 MATERIALS AND METHODS

### 2.1 Bioinformatics analysis

We used R language for data analysis and visualization. "RTCGA.miRNASeq" and "RTCGA. Clinical" download the miRNA expression profile raw data and corresponding clinical records of KIRC from TCGA. It included a total of 521 tumor samples and 72 normal tissue samples. “Deseq2,” "edgeR," and "limma" were performed to identify the differentially expressed miRNA (DEMis). *P* < 0.05 and |logFC| ≥ 1 were treated as the cutoff value. “Survival” performed survival analysis.

### 2.2 Cell culture

The human KIRC cell lines: 786‐O, A498, and human normal renal tubular epithelial cell line: HK‐2 were acquired from China Medical University (Shenyang, China). HK‐2 was cultured in Keratinocyte medium with 1% keratinocyte growth supplement (KGS, ScienCell). 786‐O and A498 were cultured in Dulbecco’s modified Eagle’s medium with 10% fetal bovine serum (DMEM, Gibco).

### 2.3 Ethics approval and consent to participate

We obtained 40 tumor samples and matched non‐tumor tissue samples from KIRC patients undergoing surgical resection at the Liaoning Province Cancer Hospital &Institute in 2019. All specimens were pathologically confirmed as KIRC. The Ethics Committee approved this study of Liaoning Cancer Hospital &Institute, and patients signed informed consent before surgery.

### 2.4 RT‐PCR

The total RNA from cells was isolated using Trizol reagent (15596018, Invitrogen). The synthetization of complementary DNA was performed in the PrimeScript RT reagent Kit (RR047A) from TaKaRa. RT‐qPCR test was constructed in Mx3000P real‐time PCR system (Agilent) using Quant one step qRT‐PCR Kit (FP303, TIANGEN) according to the below condition: 95°C pre‐denaturation for 10 min, then followed by a total of 40 cycles, denaturation (95°C, 15 s), and annealing (60°C, 1 min). The mRNA expressions of target genes were analyzed by the 2‐ΔΔCt method 18, with U6 as control. The sequences are exhibited in Supplementary Table S1.

### 2.5 Western blotting

Proteins were isolated from the KIRC cells using RIPA lysis buffer and used for Western blot analysis. Specific antibodies and the secondary antibody anti‐mouse (1:5000; GE Healthcare, NA9310) were used for the detection of cellular levels of proteins. Blots were developed using Clarity Western ECL Substrate (Bio‐Rad) and imaged. The information on antibodies was shown in Supplementary Table S2.

### 2.6 Cell transfection

KIRC cells (1 × 10^4^ cell/ml) cultured in 96‐well plates were transfected with recombinant plasmids in accordance with Lipofectamine 3000 Transfection Kit (L3000001, Invitrogen). After 24 h of cell transfection, total RNA was extracted to detect the transfection efficiency of plasmids using RT‐PCR.

### 2.7 Cell Counting Kit‐8 (CCK‐8)

CCK‐8 kit (C0037) was purchased from Beyotime to detect the cell viability in KIRC cells. The cells were sorted in 96‐well plates (1  ×  10^5^ cells per well). After being cultured routinely for hours, we added10 μl of CCK‐8 reagent to culture well. Then, the KIRC cells were continuously cultivated for 2 h. Lastly, Microplate Absorbance Reader (E0228) from Beyotime was employed to detect the absorbance (450 nm).

### 2.8 5‐ethynyl‐2’‐deoxyuridine (EdU) labeling assay

5‐ethynyl‐2’‐deoxyuridine (EdU) assays were also used to investigate the cell growth capacity. KIRC cells were placed on 96‐well plates (0.2 million cells/ml per well) and adhered overnight. Cells were then incubated with EdU for 2 h with 100 μl per well and fixed using 4% paraformaldehyde for 30 min after transfection. *In vitro* imaging dyeing was conducted by the Cell Light^™^ EdU Apollo^®^ 488 Kit (RiboBio) as per established protocol.

### 2.9 Transwell assay for cell invasion

Transwell chamber (3422, Corning) pre‐coated with Matrigel (354230, BD) was inserted into a 24‐well culture plate. The KIRC cells suspension and the medium containing 1% FBS were mixed and added to the upper chamber, and the medium containing 20% FBS was added to the 24‐well plate and cultured for 24 h. Subsequently, after being fixed with methanol, the cells on the membrane were stained with 0.1% crystal violet solution (C8470, Solarbio). The cells on the membrane were photographed under a BX53M microscope.

### 2.10 Dual‐luciferase reporter assay

Wild and mutant reporter plasmids of SIN3A which containing a wild or mutant miR‐138‐5p binding sites were produced by GenePharma. These reporter plasmids were co‐currently transfected into 786‐O cells via Lipofectamine 3000 (Invitrogen) following the manufacture. Renilla and firefly luciferase activities in triplicate assays were assessed with Luciferase Assay Kit (Promega, E1910). The activities of the luciferase were evaluated via the dual‐luciferase reporter assay system (Promega) after 48 h.

### 2.11 Statistical analysis

GraphPad Prism 8 was adopted for analyzing data. Chi‐square test, ANOVA test, two‐tailed Student’s t test, and Kaplan‐Meier analysis were used as appropriate. The data were represented as the mean ± standard error mean deviations, and a *p* value < 0.05 was deemed to have statistical significance.

## 3 RESULTS

### 3.1 miR‐138‐5p is weak‐expressed and contribute to the poor survival of KIRC

**FIGURE 1:**
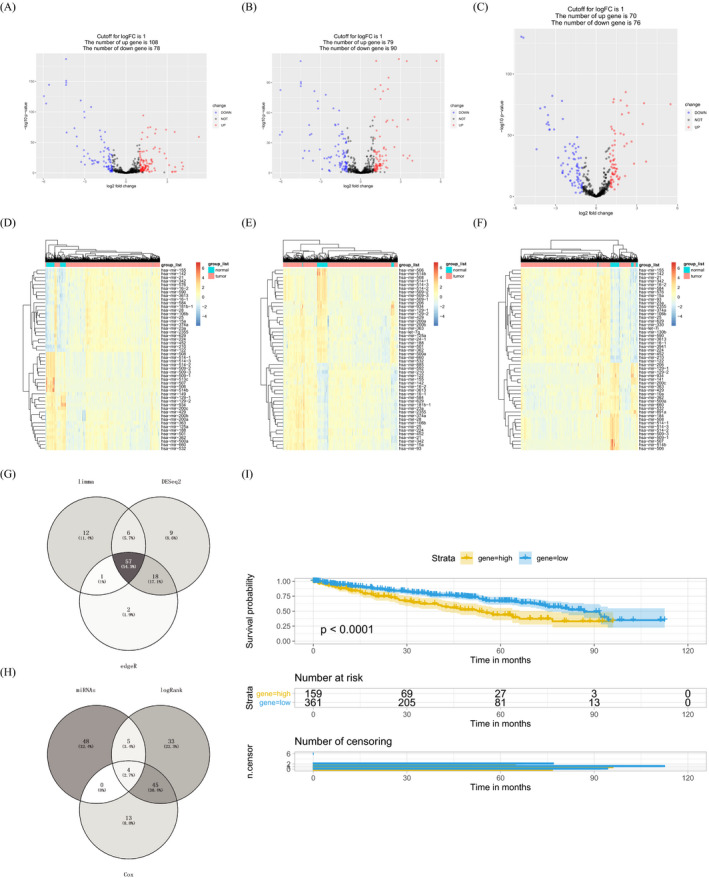
miR‐138‐5p was infertility and linked to dismal prognosis in KIRC patients. A‐C. Volcano plots exhibited the differentially expressed miRNAs by edgeR, Deseq2, and limma. D‐F. Heatmaps showed the top 50 differentially expressed miRNAs screened by edgeR, Deseq2, and limma. G. There were 57 down‐regulated miRNAs were identified by edgeR, Deseq2, and limma. H. There were 4 down‐regulated miRNAs were associated with the survival of KIRC. I. Kaplan‐Meier analyzed the correlation between miR‐138‐5p expression levels and the overall survival of KIRC in TCGA

The miRNA sequence dataset of TCGA‐KIRC included 521 KIRC samples and 72 matched para‐carcinoma tissues. The DEMis were identified by Deseq2, edgeR, and limma package (fold change ≥ 2, *p* < 0.05). There were 108 up‐regulated DEMis (uDEMis), and 78 down‐regulated DEMis (dDEMis) were identified by edgeR (Figure 1A,D). Besides, 79 uDEMis and 76 dDEMis were recognized by Deseq2 (Figure 1B,E). Moreover, 70 uDEMis and 76 dDEMis were recognized by limma (Figure 1C,F). Moreover, the heatmap showed the top 50 DEMis from each "package" analysis (Figure 1D‐F). Among them, 57 miRNAs were weak expression in all three analyses (Figure 1G). According to longrank survival (Supplementary Table S3) and Cox regression analysis (Supplementary Table S4), miR‐138‐5p and other 3 miRNAs related to KIRC survival (Figure 1H). As miR‐138‐5p was the most differentially expressed miRNAs among the four miRNAs, so we focused our attention on explaining its function in KIRC in this study (Figure 1I).

### 3.2 miR‐138‐5p inhibited cells proliferation and invasion

**FIGURE 2:**
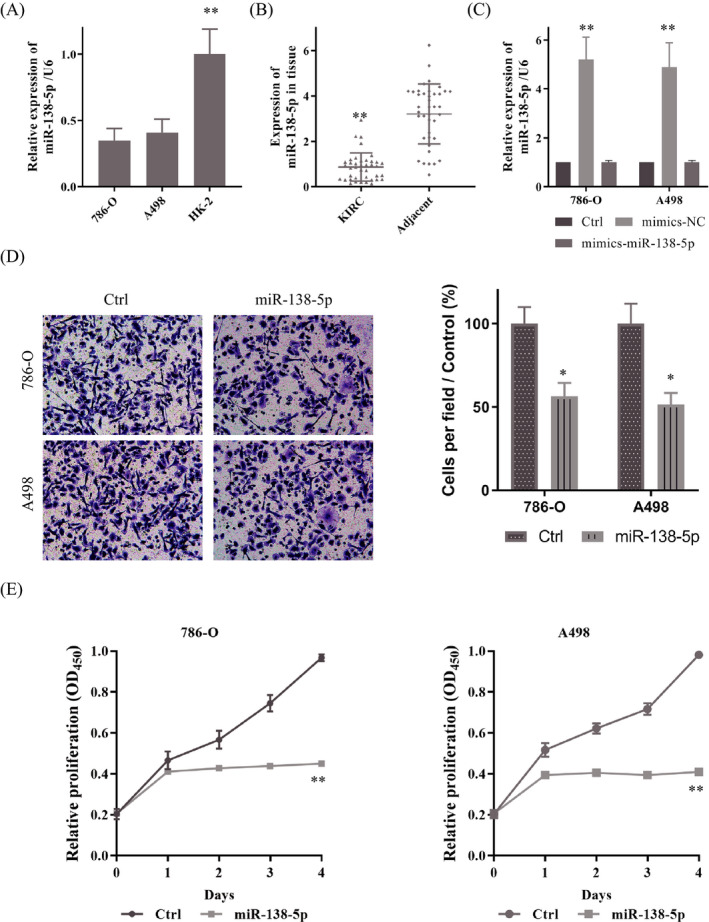
miR‐138‐5p was weak‐expressed and inhibited proliferation and invasion of KIRC. A. A. RT‐PCR showed miR‐138‐5p was weak‐expressed in KIRC cells compare to it in HK‐2. B. miR‐138‐5p expression in KIRC tissues and the adjacent normal tissues determined by RT‐PCR. C. The mimics of miR‐138‐5p up‐regulated the expression of miR‐138‐5p. D. The overexpressed miR‐138‐5p inhibited cell invasion. E, F. CCK‐8 assay indicated miR‐138‐5p could inhibit cells proliferation. Data are shown as mean ±SD, *n* = 3. Student's t test assesses statistical data significance. *p < 0.05, **p < 0.01

We detected the expression of miR‐138‐5p in KIRC cells and samples. As presented in Figure 2A, miR‐138‐5p were markedly declined in 786‐O and A498 relative to HK‐2. Besides, compared with 40 adjacent normal tissues, miR‐138‐5p were expressed at the low level in 40 matched KIRC tumor tissues (Figure 2B). We then transfected the mimics of miR‐138‐5p into 786‐O and A498; the expression of miR‐138‐5p increased sharply (Figure 2C). Transwell assay and CCK‐8 assay were used to observe the effect of miR‐138‐5p on KIRC malignant behaviors. Up‐regulated miR‐138‐5p impeded invasion ability of cells (Figure 2D). Furthermore, the CCK‐8 suggested that the up‐regulated miR‐138‐5p could suppress proliferation notably (Figure 2E).

### 3.3 miR‐138‐5p directly binds to SIN3A

**FIGURE 3:**
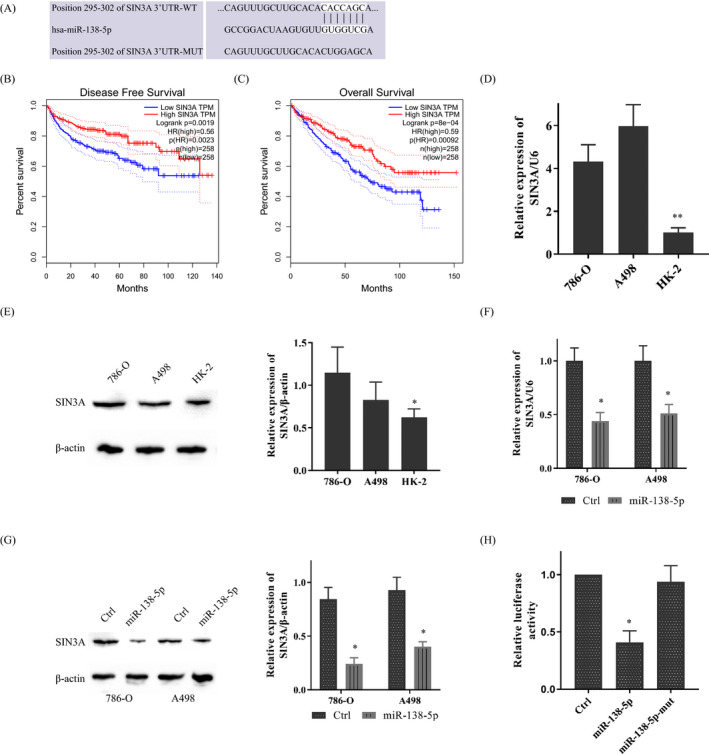
miR‐138‐5p could bind SIN3A directly. A. TargetScan predicted binding sites of miR‐138‐5p and SIN3A. B, C. SIN3A was associated with the poor DFS and OS of KIRC in TCGA. D, E. SIN3A was overexpressed in KIRC cells at both transcriptional and protein levels. F, G. miR‐138‐5p could limit the expression of SIN3A at both transcriptional and protein levels. H. Luciferase activities were measured in 786‐O cells co‐transfected with luciferase reporter containing SIN3A and the mimics of miR‐138‐5p or mutant. Data are shown as mean ±SD, *n* = 3. Student's t test assesses statistical data significance. *p < 0.05, **p < 0.01

We further explored the downstream potential molecular target. First, prediction on TargetScan, picTar, PITA, and miRanda suggested SIN3A was a potential biological target mRNA of miR‐138‐5p (Figure 3A). Analyzed the TCGA clinical data, we found that the highly expressed SIN3A was associated with the poor disease‐free survival (DFS) and overall survival (OS) in KIRC (Figure 3B,C). After that, SIN3A expression in KIRC cells was measured. RT‐PCR and Western blot showed that SIN3A was highly expressed in the KIRC cell lines compared to that in HK‐2 cells (Figure 3D,E).

Moreover, following miR‐138‐5p mimic administration, the SIN3A expression in KIRC cells was notably inhibited at both transcriptional and protein levels (Figure 3F,G). And then, a dual‐luciferase reporter assay was used to confirm the predictive result further. Data showed that the overexpression of miR‐138‐5p reduced the luciferase activity of SIN3A reporter, while had no effect on the luciferase activity of miR‐138‐5p‐mut in 786‐O (Figure 3H).

### 3.4 miR‐138‐5p constrained proliferation and invasion of KIRC by targeting SIN3A

**FIGURE 4:**
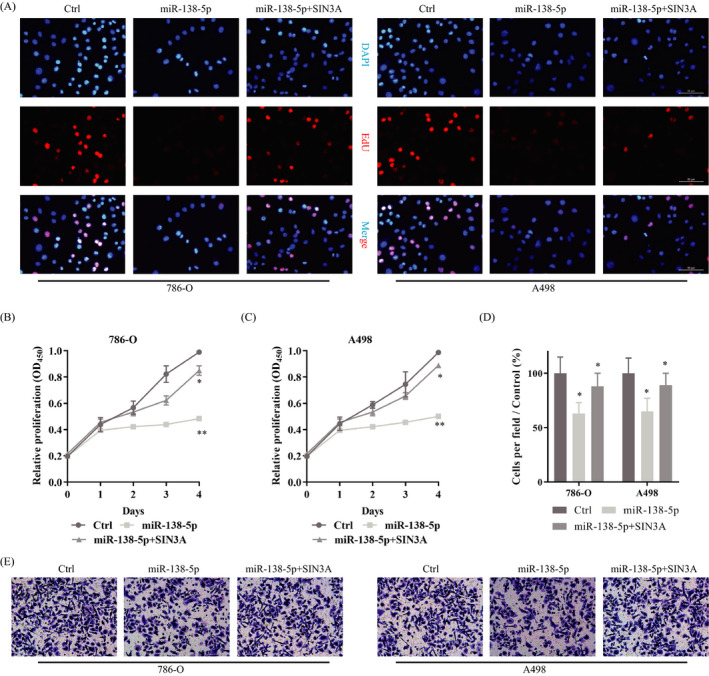
miR‐138‐5p impeded KIRC proliferation and invasion by target SIN3A. A. EdU assay confirmed miR‐27b‐3p could inhibit cell proliferation of KIRC, and the up‐regulated SIN3A could reverse this phenomenon. B, C. Proliferation of KIRC cells measured by CCK‐8 method: Overexpression of miR‐138‐5p inhibited the proliferation. On this basis, up‐regulated SIN3A could reverse this phenotype and promote the malignant proliferation of cells. D. Up‐regulated miR‐138‐5p could inhibit the invasion of KIRC, and the overexpressed SIN3A could reverse it

As explained above, miR‐138‐5p suppressed KIRC malignancy, and it could bind SIN3A directly. Next, to further confirm whether miR‐138‐5p could exert its role in a miRNA‐mRNA‐dependent manner, rescue assays were conducted in KIRC cells. We then transfected the SIN3A vector into KIRC cells, which had been treated with miR‐138‐5p mimics already. CCK‐8 and EdU assay showed that overexpressed miR‐138‐5p hinders the proliferation of KIRC cells. At the same time, on this basis, up‐regulated SIN3A could reverse this phenotype and promote the malignant proliferation of cells (Figure 4A‐C). Similarly, although overexpressed miR‐138‐5p could inhibit cell invasion, the up‐regulated SIN3A could weaken this phenomenon (Figure 4D‐E). All of these results suggested that miR‐138‐5p might regulate KIRC proliferation and invasion by targeting SIN3A. The empty plasmid+ miR‐138‐5p was set as control.

### 3.5 miR‐138‐5p suppressed proliferation and invasion of KIRC via Notch signaling pathway

**FIGURE 5:**
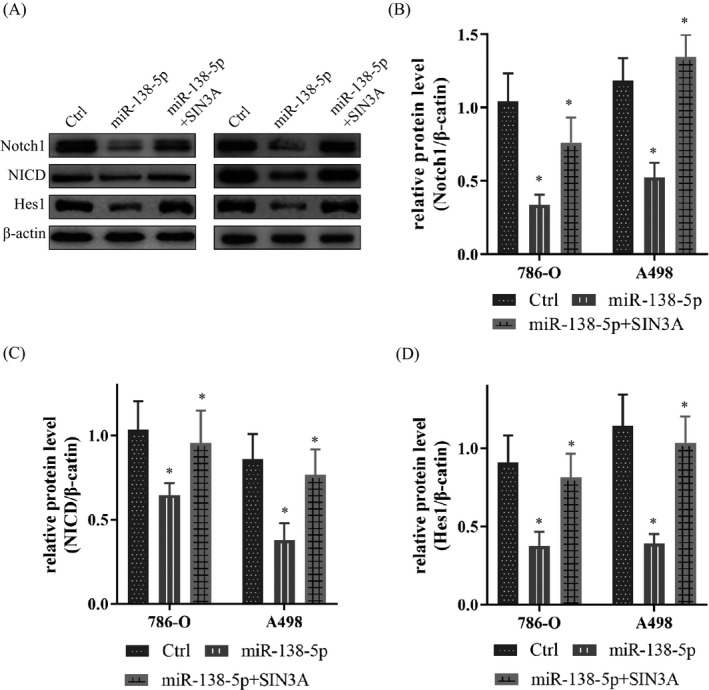
miR‐138‐5p and SIN3A regulated cell proliferation and invasion via activating the Notch signaling pathway. A. Western blot showed the protein levels of Notch1, NICD, and Hes1 in KIRC cells. B. The up‐regulated miR‐138‐5p could impede the expression of Notch1, and SIN3A could promote it. C. miR‐138‐5p could inhibit the expression of NICD, and SIN3A could reverse it. D. Hes1 could be limit by miR‐138‐5p and facilitate by SIN3A. Data are shown as mean ±SD, *n* = 3. Student's t test assesses statistical data significance. *p < 0.05

As a highly conserved signaling pathway, the Notch signaling pathway functions in cell fate decisions and widely involved in the occurrence and development of cancers.^12^, ^13^ Besides, SIN3A played an active role in the regulation of Notch signaling.^14^ We wondered whether miR‐138‐5p and SIN3A influenced the proliferation and invasion of KIRC through Notch signaling pathway. Then, we tested the biomarkers of the Notch pathway. With the increase of miR‐138‐5p, neither Notch1 (Figure 5A,B), NICD (Figure 5A,C), nor Hes1 (Figure 5A,D) was all decreased. However, the up‐regulated SIN3A increased the expression of Notch1, NICD, and Hes1 (Figure 5). It indicated miR‐138‐5p/SIN3A participates in the activation and regulation of the Notch pathway through miRNA‐mRNA mode. This pattern, in turn, affected KIRC cell proliferation and invasion.

## 4 DISCUSSION

Kidney cancer is a health‐threatening malignancy worldwide, and more than 1,000,000 new cases are diagnosed each year.^15^ As the most common pathological type of renal cancer, there is a global effort to explore genomic and epigenomic heterogeneity of KIRC to develop new specificity and sensitivity predictors and/or prognostic markers based on this to find better solutions to this health problem.^16^

With the continuous discovery of the function of miRNAs, evidence confirmed that miRNAs were abnormally expressed in KIRC and participate in the regulation of cancer progression. Some miRNAs are considered molecular targets for KIRC therapy. With the expansion of research on miRNAs function, there is growing evidence that a range of miRNAs are abnormally expressed in cancer and are involved in regulating cancer progression. Increasingly, miRNAs have been identified as biomarkers for KIRC therapy.^17^, ^18^

To avoid bias, in this study, miRNA transcriptome data of KIRC were downloaded from TCGA. Through bioinformatics analysis, including differential expression analysis and survival analysis, we found that weak expression of miR‐138‐5p was an essential factor associated with the poor prognosis of KIRC. miR‐138‐5p was weak‐expressed in some kinds of cancers: It acted as a tumor suppressor gene in colorectal cancer to imped cell proliferation, and aerobic glycolysis served as a competing endogenous RNA.^19^ miR‐138‐5p participated in the inhibition of astragalus polysaccharides to prostate cancer.^20^ miR‐138‐5p hamper DHA metabolite and lung cancer metastasis by targeting FOXC1.^21^ Therefore, we believed that miR‐138‐5p might inhibit tumor development, that is, overexpressed miR‐138‐5p might have anti‐tumor effects. Through the detection of miR‐138‐5p in tissue samples and cells, we found that miR‐138‐5p was weak expression in them. This confirmed our suspicions. We then transfected the mimics into tumor cells to identify the molecular mechanisms of miR‐138‐5p. CCK‐8 and EdU results clarified that miR‐138‐5p could inhibit cell proliferation. Transwell suggested miR‐138‐5p could significantly reduce transmembrane KIRC cells. These results suggested that miR‐138‐5p had a significant inhibitory effect on cell proliferation and invasion.

SIN3A is a highly conserved transcriptional regulatory protein. It contains paired amphipathic helix domains that play an essential role in protein‐protein interactions.^22^ There is also a histone‐interacting domain in SIN3A. It helps SIN3A recruit a core complex that involves histone binding proteins, stabilizing proteins, and class I HDAC enzymes.^23^ It was involved in STAT3‐related transcriptional activation and regulation, and the STAT3‐SIN3A axis might be a potential target for tumor therapy.^24^ In the experiment, we found and proved that miR‐138‐5p could directly bind to the 3'‐UTR of SIN3A and participated in the transcription of SIN3A. Rescue assay further demonstrated that miR‐138‐5p's role in KIRC proliferation and invasion was realized through SIN3A.

As a conserved and famous signaling pathway, the Notch pathway determines cell fate to some extent. Changes in the microenvironment and gene expression lead to dysregulate of Notch signaling, which then plays an intricate role in carcinogenesis or tumor inhibition in different tumors. Moreover, it has widely crosstalk with other oncogenes and signaling pathway^25^ and participate in the cancer proliferation and invasion.^26^, ^27^ Notch pathway in mammals includes four receptors (Notch 1–4) and five ligands (DLL 1–3, Jag 1–2). Once the receptor and ligand bind, the release of the intracellular domain (NICD) of Notch activates downstream genes such as Hes.^28^ In this study, we found that overexpressed miR‐138‐5p inhibited the expression of Notch1, NICD, nor Hes1 and inhibited the activation of the Notch pathway. Furthermore, consistent with the previous rescue assay results, the overexpressed SIN3A could reverse the effect of miR‐138‐5p and promoted the Notch pathway's activation.

In conclusion, our data confirmed that the up‐regulation of miR‐138‐5p could significantly repress KIRC proliferation and invasion via suppressing SIN3A production and subsequently regulating the Notch pathway. Consequently, our discoveries gave practical evidence to detect new‐found target for therapeutic treatment in KIRC evolution.

## 5 ETHICS APPROVAL AND CONSENT TO PARTICIPATE

The study was reviewed and approved by the Faculty of Science Ethics Committee at Liaoning Cancer Hospital & Institute (Cancer Hospital of China Medical University) (20181227)

## CONFLICT OF INTEREST

The authors declare that they have no conflict of interest

## AUTHOR CONTRIBUTIONS

Liu Y performed the majority of experiments and analyzed the data and drafted the manuscript; Qu HC designed the research and conducted the molecular biology assays and analyzed the data.

## Supporting information

Table S1

Table S2

Table S3

Table S4

## Data Availability

The datasets used and/or analyzed during the present study are available from the corresponding author on reasonable request.
