# Supplementary material for: miR‐138‐5p inhibits proliferation and invasion in kidney renal clear cell carcinoma by targeting SINA3 and regulation of the Notch signaling pathway
Source: J Clin Lab Anal. 2021 Sep 29;35(11):e23766. doi: 10.1002/jcla.23766 (PMC8605131; doi:10.1002/jcla.23766)
Supplement: Supplementary file 1 — Table S1 [file JCLA-35-e23766-s002.docx]

| Primer | Primer Sequence (5′-3′) |
| --- | --- |
| miR-138b-5p | F: GCGAGCTGGTGTTGTGAATC |
|  | R: AGTGCAGGGTCCGAGGTATT |
| SIN3A | F: CTTCGTTAGTGGAGAGGTAG |
|  | R: GTGATGATGGCTGCTATGA |
| U6 | F: CGCTTCGGCAGCACATATAC |
|  | R: TTCACGAATTTGCGTGTCATC |

Supplement TABLE 1 Primer sequences for RT-PCR
