# Supplementary material for: miR‐138‐5p inhibits proliferation and invasion in kidney renal clear cell carcinoma by targeting SINA3 and regulation of the Notch signaling pathway
Source: J Clin Lab Anal. 2021 Sep 29;35(11):e23766. doi: 10.1002/jcla.23766 (PMC8605131; doi:10.1002/jcla.23766)
Supplement: Supplementary file 2 — Table S2 [file JCLA-35-e23766-s004.docx]

Supplement TABLE 2 Antibodies used for Western blot analysis

| Antibody | Company | Dilution |
| --- | --- | --- |
| SIN3A | Abcam | 1:1000 |
| Notch1 | Abcam | 1:500 |
| NICD | Abcam | 1:500 |
| Hes1 | Abcam | 1:500 |
| β-actin | ProteinTech | 1:8000 |
